# Avian Influenza Risk Perception and Preventive Behavior among Traditional Market Workers and Shoppers in Taiwan: Practical Implications for Prevention

**DOI:** 10.1371/journal.pone.0024157

**Published:** 2011-09-02

**Authors:** Pei-Chun Kuo, Jiun-Hau Huang, Ming-Der Liu

**Affiliations:** 1 Institute of Health Policy and Management, College of Public Health, National Taiwan University, Taipei, Taiwan; 2 Department of Public Health, College of Public Health, National Taiwan University, Taipei, Taiwan; 3 College of General Education, Hungkuang University, Taichung, Taiwan; University of Hong Kong, Hong Kong

## Abstract

**Background:**

Avian influenza (AI) can be highly pathogenic and fatal. Preventive behavior such as handwashing and wearing face masks has been recommended. However, little is known about what psychosocial factors might influence people's decision to adopt such preventive behavior. This study aims to explore risk perception and other factors associated with handwashing and wearing face masks to prevent AI.

**Methodology/Principal Findings:**

An interviewer-administered survey was conducted among 352 traditional market workers and shoppers in Taiwan between December 2009 and January 2010. Factors associated with the recommended AI preventive behavior (i.e., when in a traditional market, wearing a face mask and also washing hands after any contact with poultry) included: having correct knowledge about the fatality rate of AI (adjusted odds ratio [AOR] = 4.18), knowing of severe cases of AI (AOR = 2.13), being informed of local AI outbreaks (AOR = 2.24), living in northeastern Taiwan (AOR = 6.01), having a senior high-school education (AOR = 3.33), and having a university or higher education (AOR = 6.86). Gender interactive effect was also found among participants with a senior high-school education, with males being less likely to engage in the recommended AI preventive behavior than their female counterparts (AOR = 0.34).

**Conclusions/Significance:**

Specific information concerning AI risk perception was associated with the recommended AI preventive behavior. In particular, having correct knowledge about the fatality rate of AI and being informed of severe cases and local outbreaks of AI were linked to increased AI preventive behavior. These findings underscore the importance of transparency in dealing with epidemic information. These results also have practical implications for prevention and policy-making to more effectively promote the recommended AI preventive behavior in the public.

## Introduction

A total of 520 cases of avian influenza (AI) had been reported around the world, resulting in 307 deaths, with a case fatality rate of 59.0% [1]. The AI virus has been found in domestic poultry (e.g., chickens, ducks, geese), wild birds [2], waterfowl and shorebirds [3]. An extensive review indicated that the AI virus could be transmitted through direct contact with infected poultry, including holding diseased or dead poultry, slaughtering, defeathering, or preparing sick poultry for cooking [4].

Taiwan experienced sporadic AI outbreaks in 2004 and most recently in 2009, including major local outbreaks in Kaohsiung (a region located in southern Taiwan) in 2008. Limited information released in government reports [5] later confirmed that these outbreaks were caused by H5N2 virus. Although there had not been any highly pathogenic avian influenza (HPAI) H5N1 cases reported in Taiwan, yet considering the geographical location of Taiwan being an important stopover for migrating birds [6] and previous H5N1 outbreaks in neighboring Asian countries such as Thailand and China [7], plus increasing travel and direct transportation links with other countries, Taiwan is at risk for HPAI outbreaks as well. The most worrisome scenario is that human-to-human transmissions may begin to take place if there is a change in the viral genome [8], and according to a relatively conservative estimate by the World Health Organization (WHO), such transmissions may cause 2 million to 7.4 million deaths [9].

In Taiwan, people have the habit of shopping at traditional markets for live poultry [10], and such traditional markets with live chicken for sale provide a possible AI viral reservoir [11], thereby placing traditional market workers and shoppers at risk for contracting AI. Poultry are usually selected and purchased by shoppers while they are still alive and slaughtered on site as shoppers in Taiwan commonly believe that live poultry preserves the freshness [11]. During the purchasing process, shoppers may come into contact with live poultry, thereby exposing shoppers to risk of contracting AI, and thus it is important that shoppers wash their hands after any contact with poultry. Normally, a poultry vendor would not sell other foods; however, in a traditional market, a poultry stand could be adjacent to any other food stands without any partitions separating them. Notably, AI viruses can also be transmitted to humans through the particles raised up by the movement of the poultry [12], and therefore, regardless of whether the shoppers are purchasing live poultry, they are advised to wear a face mask while in a traditional market. Given the modes of AI transmission as described above, WHO [13] and Taiwan Centers for Disease Control (Taiwan CDC) [14] both recommended washing hands after any contact with poultry and using a face mask when coming into contact with poultry to prevent AI infection and its spread. Handwashing [15], [16] and wearing face masks [17] have been documented as effective preventive measures against respiratory disease in community settings. In addition, these two preventive behaviors are relatively easy to practice. Taken together, both handwashing and wearing face masks could be a cost-effective way of preventing AI in the general public. Therefore, an enhanced understanding of the factors associated with AI preventive behavior could inform renewed prevention efforts to more effectively promote the recommended preventive behavior in different target populations.

Previously, studies have been conducted in various countries in regard to AI. For example, a study in Turkey investigated AI knowledge and anticipated attitudes in the general population; however, this cross-sectional survey only examined the anticipated preventive measures rather than actual preventive behaviors [18]. Another study in Italy focused merely on poultry workers, exploring the relationships of their knowledge, attitudes, and compliance with precautions at work, such as self-reported use of face masks and gloves [19]. An earlier study reported its findings in a letter to the editor, presenting regional differences in AI knowledge, risk perceptions, and AI-related behavior changes among Laotians after HPAI outbreaks [20]. Further, a telephone survey in the Hong Kong general population examined participants' AI risk perception in relation to their live chicken purchasing behavior [21]. Similarly, a study in Taiwan conducted by marketing and business management researchers assessed consumer knowledge and risk perceptions of AI in association with chicken consumption behavior, instead of AI preventive behavior under the threat of AI [11].

However, relatively little is known about the recommended handwashing behavior, especially in combination with face mask wearing behavior, to prevent AI. Therefore, considering the modes of AI transmission taking place at traditional markets in Taiwan as reviewed earlier, the current study aimed to estimate the prevalence of the recommended AI preventive behavior (i.e., when in a traditional market, wearing a face mask and washing hands after any contact with poultry) among traditional market workers and shoppers in Taiwan and examined their knowledge and risk perception in relation to AI preventive behavior.

## Methods

### Data Collection

Participants of this cross-sectional study were market workers and shoppers in traditional markets located in northeastern and central Taiwan. As described in the Introduction, there were major AI outbreaks in Kaohsiung (in southern Taiwan), and therefore, the current study aimed to explore the risk perception about AI, as well as the recommended AI preventive behavior, among traditional market workers and shoppers in central and northeastern Taiwan, where there have not been reported AI outbreaks. Accordingly, two traditional markets were selected in central Taiwan and two others were selected in northeastern Taiwan. Since this study sought to examine simultaneously both groups of participants (i.e., traditional market workers and shoppers), we decided to interview market workers and shoppers with a one-to-one ratio. With this particular purpose in mind, purposive sampling was employed in traditional markets: market workers were interviewed by trained interviewers during their breaks; because there were far more market shoppers than workers, market shoppers were randomly selected for an interview at the market entrance if they happened to step into the market when an interviewer just became available to conduct a survey after completing the previous interview. The interviewer-administered survey using a structured questionnaire was conducted in December 2009 through January 2010. A total of 352 anonymous interviews were completed with a response rate of 95.1%. Each interview was conducted by a trained interviewer and took the participant 5–10 minutes to complete the questionnaire. Traditional market workers and shoppers were first approached and informed of the study's goals and procedures by our interviewers to obtain verbal informed consent before each interview. Every attempt made to approach potential participants, be it successful or unsuccessful, was documented in order to calculate the response rate. We also provided a gift worth approximately US $1 as incentives to increase the response rate. The study protocol and verbal consent procedure were reviewed and approved by the Institutional Review Board of the National Taiwan University College of Public Health.

### Measures

The survey collected sociodemographic information such as the participant's age, gender, region of residence, education, and whether the participant was a market worker (yes/no). This study also classified each participant's risk of AI by type of work: 1) nonmarket worker (i.e., shopper), 2) low risk market worker (e.g., flower vendor, vegetable vendor, cleaner, administrative staff), 3) medium risk market worker (e.g., pork vendor, beef vendor, seafood vendor, mutton vendor, cooked poultry vendor), and 4) high risk market worker (e.g., chicken butcher, chicken vendor, poultry organ vendor). It is noteworthy that the AI risk classification above takes into account the nearness in space to live poultry. For example, meat and seafood stands are normally located in the same section as poultry stands in a traditional market, and hence are closer to poultry vendors than are flower and vegetable vendors. As such, pork vendors, beef vendors, etc. are classified as medium risk market workers, whereas flower vendors and vegetable vendors are classified as low risk market workers.

Further, this study assessed the participant's knowledge about AI such as its transmissibility and fatality rate (Table S1) [31]. The survey also evaluated the participant's risk perception of AI, including whether the participant anticipated an AI epidemic in Taiwan, whether the participant knew about severe cases of AI, and whether the participant knew about AI outbreaks in Kaohsiung (a region located in southern Taiwan). The variables regarding knowledge and risk perception of AI were binary (e.g., yes/no, correct/incorrect). The outcome measure of recommended AI preventive behavior was assessed by asking: “When you are in a traditional market, do you wear a face mask and also wash your hands after any contact with poultry (yes/no)?” Notably, each participant's response to this behavioral outcome measure was validated by the interviewer through direct observation in regard to the face mask wearing aspect of the preventive behavior. In other words, since all interviews were conducted in traditional markets, only participants who were wearing a face mask at the time of the interview would be coded as adopting the recommended AI preventive behavior as defined in this study. Several theoretical models have been used for understanding protective health behaviors, including the Health Belief Model (HBM) [22]–[24]. The risk perception measures in this study were derived from theoretical constructs of the HBM, which posits that risk perceptions such as perceived severity and perceived susceptibility are associated with adoption of health-related behaviors [25]. This model has also been used in a recent AI study [26].

### Statistical Analysis

Descriptive statistics were first examined for sociodemographic data. Sociodemographic variables, knowledge, and risk perception of AI were cross-tabulated with AI preventive behavior, and χ^2^ comparisons were performed to test for group differences between participants who practiced and those who did not practice the recommended AI preventive behavior. T-test was used for comparing the ages of participants who practiced the recommended AI preventive behavior and those who did not. Then, variables with significant χ^2^ or t-test results were included as candidates in subsequent stepwise logistic regression modeling. The final model was adjusted for age and gender as control variables. All statistical analyses were carried out with SPSS (version 17) and *P*<.05 indicated statistical significance.

## Results

### Sociodemographic Characteristics of Participants

The sociodemographic characteristics of the participants (n = 352) are as follows: the mean age was 43.9 years; 62.5% were female; 59.4% lived in central Taiwan; 18.2% had a degree from university or above, and 41.2% had a senior high-school diploma. About half (49.7%) of the participants were shoppers; 22.7%, 18.2%, and 9.4% were market workers at low, medium, and high risk for AI, respectively. Slightly more than half (52.6%) of the participants adopted the recommended AI preventive behavior.

### Knowledge and Risk Perception of AI

This study found that 44.3% and 26.4% of the participants had correct knowledge about AI transmissibility and AI fatality rate, respectively. Further, 44.0% of the participants anticipated an AI epidemic in Taiwan, 73.9% knew about AI severe cases, and 38.9% knew about AI outbreaks in Kaohsiung.

### Associations with AI Preventive Behavior

Table 1 shows the associations of sociodemographic characteristics and AI risk perception with the recommended AI preventive behavior. Younger participants were more likely than older participants (mean age = 42.0 vs. 45.6, t = 3.00, p = .003), females were more likely than males (50.0% vs. 43.2%, χ^2^ = 1.54, df = 1, p = .22), and participants who lived in northeastern Taiwan were more likely than those in central Taiwan (62.9% vs. 36.8%, χ^2^ = 23.19, df = 1, p<.0001) to practice the recommended AI preventive behavior. Furthermore, participants with a university or higher degree (70.3%) were the most likely to adopt the AI preventive behavior, followed by those with a senior high-school diploma (53.8%) and those with a junior high-school or less education (30.8%) (χ^2^ = 37.71, df = 2, p<.0001). Finally, compared with their counterparts, participants who had correct knowledge about AI fatality rate (68.8% vs. 39.8%, χ^2^ = 23.16, df = 1, p<.0001), who anticipated an AI epidemic in Taiwan (54.8% vs. 41.6%, χ^2^ = 6.08, df = 1, p = .01), who knew about severe cases of AI (55.0% vs. 26.1%, χ^2^ = 22.78, df = 1, p<.0001), and who knew about AI outbreaks in Kaohsiung (63.5% vs. 37.2%, χ^2^ = 23.20, df = 1, p<.0001) were more likely to practice the recommended AI preventive behavior.

**Table 1:** Associations of sociodemographic characteristics and AI risk perception with the recommended AI preventive behavior (n = 352).^a^

	Adopted AI preventive behavior^b^					
	No		Yes		χ^2^ (df)	p value
Variable	No.	Row (%)	No.	Row (%)		
Gender					1.54 (1)	.22
Male	75	(56.8)	57	(43.2)		
Female	110	(50.0)	110	(50.0)		
Region of residence					23.19 (1)	<.0001
Northeastern Taiwan	53	(37.1)	90	(62.9)		
Central Taiwan	132	(63.2)	77	(36.8)		
Education					37.71 (2)	<.0001
Junior high-school or below	99	(69.2)	44	(30.8)		
Senior high-school	67	(46.2)	78	(53.8)		
University or above	19	(29.7)	45	(70.3)		
Market worker					0.72 (1)	.40
Yes	97	(54.8)	80	(45.2)		
No	88	(50.3)	87	(49.7)		
Risk of AI by type of market work					2.16 (3)	.54
Shopper (non-market worker)	88	(50.3)	87	(49.7)		
Low risk	41	(51.3)	39	(48.8)		
Medium risk	35	(54.7)	29	(45.3)		
High risk	21	(63.6)	12	(36.4)		
Knowledge about AI transmissibility					1.54 (1)	.22
Correct	76	(48.7)	80	(51.3)		
Incorrect	109	(55.6)	87	(44.4)		
Knowledge about AI fatality rate					23.16 (1)	<.0001
Correct	29	(31.2)	64	(68.8)		
Incorrect	156	(60.2)	103	(39.8)		
Anticipated an AI epidemic in Taiwan					6.08 (1)	.01
Yes	70	(45.2)	85	(54.8)		
No	115	(58.4)	82	(41.6)		
Knew about AI severe cases					22.78 (1)	<.0001
Yes	117	(45.0)	143	(55.0)		
No	68	(73.9)	24	(26.1)		
Knew about AI outbreaks in Kaohsiung					23.20 (1)	<.0001
Yes	50	(36.5)	87	(63.5)		
No	135	(62.8)	80	(37.2)		
	**Mean**	**SD**	**Mean**	**SD**	**t**	**p value**
Age (year)	45.6	(11.3)	42.0	(11.0)	3.00	.003

a AI, avian influenza; df, degrees of freedom; SD, standard deviation.

b Defined by: “When you are in a traditional market, do you wear a face mask and also wash your hands after any contact with poultry (yes/no)?”

### Multivariate Analysis for Covariates of AI Preventive Behavior

Table 2 presents the final multivariate logistic regression model for covariates of AI preventive behavior. Participants who lived in northeastern Taiwan were 6 times as likely as those in central Taiwan to practice the recommended AI preventive behavior (adjusted odds ratio [AOR] = 6.01, 95% confidence interval [95% CI] = 3.40–10.61). Gender did not have a statistically significant effect; however, male gender was found to interact with senior high-school education (AOR = 0.34, 95% CI = 0.12–0.98). Hence, females with a senior high-school diploma were more than 3 times as likely (AOR = 3.33, 95% CI = 1.56–7.07), and participants with a university or higher degree were nearly 7 times as likely (AOR = 6.86, 95% CI = 2.60–18.06) to adopt the AI preventive behavior, compared with their counterparts with a junior high-school or less education. Further, participants who had correct knowledge about AI fatality rate were more than 4 times as likely (AOR = 4.18, 95% CI = 2.25–7.75), those who knew about AI severe cases were approximately 2 times as likely (AOR = 2.13, 95% CI = 1.13–3.99), and those who knew about AI outbreaks in Kaohsiung were more than 2 times as likely (AOR = 2.24, 95% CI = 1.28–3.92) to practice the AI preventive behavior, compared with their counterparts.

**Table 2:** Multivariate logistic regression model for covariates of AI preventive behavior (n = 352).^a^

Variable	AOR	95% CI
Age (year)	1.01	0.98–1.04
Gender		
Male	1.73	0.85–3.51
Female	1.00^b^	
Region of residence		
Northeastern Taiwan	6.01	3.40–10.61
Central Taiwan	1.00^b^	
Education		
Junior high-school or below	1.00^b^	
Senior high-school	3.33	1.56–7.07
University or above	6.86	2.60–18.06
Knowledge about AI fatality rate		
Correct	4.18	2.25–7.75
Incorrect	1.00^b^	
Knew about AI severe cases		
Yes	2.13	1.13–3.99
No	1.00^b^	
Knew about AI outbreaks in Kaohsiung		
Yes	2.24	1.28–3.92
No	1.00^b^	
Senior high-school education by male gender^c^	0.34	0.12–0.98

a AI, avian influenza; AOR, adjusted odds ratio; CI, confidence interval.

b Reference category.

c Interaction between senior high-school education and gender.

## Discussion

### Knowledge of AI Fatality Rate and Risk Perception

An earlier study reported that greater knowledge of AI (i.e., knowing correctly the modes of AI transmission, occupational groups at risk for AI infection, and proper AI preventive measures) among poultry workers was associated with increased odds of adopting preventive measures, including wearing protective clothing and face masks [19]. Adding to the literature, our study further found that, compared with participants who misperceived that AI fatality rate is lower than that of pandemic H1N1, those with correct knowledge were more than 4 times as likely to practice the recommended AI preventive behavior. Another study, conducted among adults in the general population, also reported that participants who had correct knowledge about AI were more likely to practice AI preventive behavior [27]. While these prior studies also identified knowledge of AI, such as modes of AI transmission, to be a significant factor for increased preventive behavior, our study discovered that to promote the recommended AI preventive behavior, it is crucial to inform the public specifically of the AI fatality rate. In addition, unlike most previous research [18]–[20] which focused on poultry workers or the general public, this study expanded this line of research by examining AI preventive behavior and related factors among traditional market workers and shoppers.

### Importance of Transparency: AI Severe Cases and Local Outbreaks

This study also found that participants with greater risk perception of AI (i.e., those who knew about AI severe cases and those who knew about AI outbreaks in Kaohsiung) were more likely to practice the recommended AI preventive behavior. The greater adoption of precautionary measures among these participants with higher risk perception in the current study could be explained by their possibly elevated anxiety levels as posited in an earlier study [28]. These findings further underscore the importance of transparency in dealing with epidemic information, specifically, AI severe cases and local outbreaks, if any, as in this study. Accordingly, future public service announcements or pandemic control initiatives should consider disseminating the aforementioned specific information to the public in the face of an AI epidemic.

### Regional Variation and Cross-Cultural Differences

Participants living in northeastern Taiwan were found to be much more likely to practice the recommended AI preventive behavior than those living in central Taiwan in the current study. Such regional differences in preventive behavior were also reported in a Laotian population [20] and were attributed to different participant characteristics in urban and rural areas. In light of such findings, the present study also took into account gender, age, education, and other covariates in the multivariate regression model. However, the regional differences still remained. A possible explanation is that participants in northeastern Taiwan, which is more rural and has fewer healthcare resources than central Taiwan, may choose the relatively easy and cost-effective measures such as wearing face masks and washing hands to protect against AI. Another study in China also found differences between Guangzhou and Hong Kong in regard to participants' AI risk perception and live poultry purchase [29]; while its outcome variable of interest was not AI preventive behavior, regional differences were present and were ascribed to cultural differences. Notably, the current study found that participants who lived in rural areas were more likely to practice the recommended AI preventive behavior (i.e., both face mask wearing and handwashing) than those who lived in urban areas; however, prior research has found that people living in rural areas were more likely to practice the AI risky behavior (i.e., live poultry purchase) than those living in urban areas [29]. Caution needs to be exercised in interpreting such inconsistency, because different behaviors could have different determinants, and therefore, it is possible that these two studies found inconsistent results due to the different natures of these behaviors in question. These findings suggest that future studies may investigate potential cross-national differences in AI preventive behavior, and that qualitative research is also needed to explore regional differences caused by cross-cultural differences as well as other possible causal mechanisms.

### Interactive Effect between Gender and Education

Gender differences in the practice of protective behavior against emerging infectious diseases, including SARS and AI, have been explored in prior research, although the results have been inconclusive. For example, a review article on handwashing practices during and after SARS outbreak indicated that females in general were more likely than males to adopt the protective behavior, suggesting that females might be more health conscious and risk averse, although the reported differences were not always statistically significant [16]. Similarly, in a limited number of AI studies which included gender as a variable, its effect on the AI preventive behavior was generally found to be not statistically significant [e.g.], [19,26]. On the other hand, higher education levels have repeatedly been reported to be associated with increased knowledge and intention to adopt the recommended AI preventive behavior [e.g.], [18], [19,26]. Consistent with these findings, our study also found participants with higher education levels to be more likely to practice the AI preventive behavior. Interestingly, while gender difference was not statistically significant in the current study, we found significant interactive effect between gender and education among participants with a senior high-school education, males being less likely to adopt the recommended AI preventive behavior than their female counterparts. To our knowledge, such findings have not been reported in previous research and warrant further investigations to elucidate possible mechanisms.

### Traditional Market Workers and Risk for AI

Moreover, the χ^2^ comparison in this study found an alarming pattern that market workers at higher risk for AI appeared to be less likely to adopt preventive behavior than shoppers and other market workers with lower risk for AI. Further, an ancillary analysis (data not shown) indicated that these high-risk market workers also had a significantly lower level of education, which was associated with lower compliance with recommended preventive behavior. Taken together, more attention should be paid to this group of high-risk market workers. It is worth noting that the aforementioned χ^2^ comparison of AI preventive behavior was not statistically significant, possibly owing to relatively smaller cell counts of high-risk market workers and hence reduced statistical power. Therefore, future studies may consider increasing not only the total sample size but also the number of high-risk market workers so as to confirm the above-noted pattern.

### Limitations and Future Directions

Limitations of this study include the potential reverse causality due to the cross-sectional design; however, a number of variables identified to be significantly associated with AI preventive behavior (e.g., region of residence and education) are likely to precede temporally the outcome measure, thereby lending additional support to our explanations discussed earlier. Also, combining wearing face masks and handwashing as the outcome variable without assessing them separately could be a limitation of this study because determinants of these two practices could be different. On the other hand, however, in view of the modes of AI transmission through contact and air particles, practicing both face mask wearing and handwashing behaviors could provide better protection against AI infection. Another potential limitation is that this study was not based on national data but on data from northeastern and central Taiwan; yet, regional differences were uncovered. Therefore, future studies should consider drawing a national sample to explore other possible cross-regional differences in Taiwan. In addition, cross-national comparisons may also reveal interesting differences across various countries and cultures in preventive behaviors associated with such emerging infectious diseases. Lastly, while self-efficacy (a construct borrowed from Bandura's Social Learning Theory) was not added to the HBM until 1988 [30], it has increasingly become an important construct in the HBM but was not included in our study. Hence, future research should consider measuring all other HBM constructs, including self-efficacy, when examining AI preventive behavior.

### Conclusion

In conclusion, this study found that specific information concerning AI risk perception was associated with the recommended AI preventive behavior. In particular, having correct knowledge about the fatality rate of AI, and being informed of severe cases of AI and local AI outbreaks, were linked to increased AI preventive behavior. These findings have important implications for future practice as they could inform policy-making and renewed prevention efforts to more effectively promote the recommended AI preventive behavior in the public.

## Supporting Information

Table S1 **Questions used to measure AI knowledge, risk perception, and preventive behavior.**
(DOC)
