# Supplementary material for: Avian Influenza Risk Perception and Preventive Behavior among Traditional Market Workers and Shoppers in Taiwan: Practical Implications for Prevention
Source: PLoS One. 2011 Sep 2;6(9):e24157. doi: 10.1371/journal.pone.0024157 (PMC3166308; doi:10.1371/journal.pone.0024157)
Supplement: Table S1 — Questions used to measure AI knowledge, risk perception, and preventive behavior. (DOC) [file pone.0024157.s001.doc]

**Table S1.** Questions used to measure AI knowledge, risk perception, and preventive behavior.

| **Variable** | **Question** | **Response Category** |
| --- | --- | --- |
| Knowledge | 1. Do you think AI is more transmissible than pandemic H1N1? | Yes/No (Recoded into Incorrect/Correct)^a^ |
|  | 2. Do you think AI is more fatal than pandemic H1N1? | Yes/No (Recoded into Correct/Incorrect)^a^ |
| Risk Perception | 1. Do you anticipate an AI epidemic in Taiwan? | Yes/No |
|  | 2. Did you know about severe cases due to AI infection? | Yes/No |
|  | 3. Did you know about AI outbreaks in Kaohsiung? | Yes/No |
| Prevent Behavior | When you are in a traditional market, do you wear a face mask and also wash your hands after any contact with poultry? | Yes/No |

^a^AI has lower transmissibility but a higher case fatality rate, compared with pandemic H1N1 [1,31].
